# Polyglutamine Leads to Altered Chromatin Architecture and Neurodegeneration

**DOI:** 10.1371/journal.pbio.0040070

**Published:** 2006-02-28

**Authors:** Richard Robinson

In the field of neurodegenerative disease, the so-called polyglutamine diseases hold a unique place. These nine different diseases, which include Huntington disease and several forms of spinocerebellar ataxia, involve nine different genes. But each gene carries the same mutation—an increase in the number of CAG nucleotide triplets. In the normal genes, the number of CAG units is 30 or less; in the mutated form, this grows to 35 or more. CAG codes for the amino acid glutamine, and the consequence of each mutation is to create an expanded polyglutamine tract within the protein encoded by the gene. The presence of this tract kills nerve cells, by a mechanism or mechanisms that still remain unclear.

Despite the similarity of the mutations, each disease differs in the exact set of cells it affects within the brain. This different behavior is presumed to arise from differences in the normal function of the various polyglutamine-expanded proteins, and from the cellular context in which each is expressed. But for many of these diseases, the role of the normal protein is not well understood, making it difficult to determine whether expanded polyglutamine interferes with this function. In a new study, Dominique Helmlinger, Didier Devys, and colleagues show that in spinocerebellar ataxia type 7 (SCA7), the damage done to the retina is a direct consequence of the normal protein's key role in turning on genes specifically expressed in photoreceptor cells.

The authors have previously shown that ataxin-7 (the protein made by the SCA7 gene) is a subunit in two highly related transcriptional coactivator complexes, which stimulate gene transcription by modifying the histone proteins around which DNA is wrapped. They also developed a mouse model of SCA7 in which the mutant gene is expressed only in the rod cells of the retina, leading to retinal degeneration (in humans, retinal degeneration is a prominent part of the disease). In this study, they examined the cell architecture of the degenerating rod cells, and found that the nucleus was highly swollen due to dramatically increased “decondensation” of the chromatin, or nuclear material.

Decondensation occurs when histones, which help package DNA in the nucleus, are chemically modified by acetylation (adding a two-carbon acetyl group). Acetylation opens up the chromatin, allowing the gene-transcribing machinery to gain access to genes within. Rod cell degeneration was directly correlated with the degree of decondensation, suggesting that aberrant decondensation might be the primary event leading to cell death.

Decondensation usually leads to increased gene transcription, but the authors found instead that transcription of rod-specific genes was decreased, in many cases by 90% or more. This was not due to any inability of the mutant protein to become incorporated into its two transcriptional coactivator complexes, which appeared to be unchanged. Instead, the authors show that these complexes worked too well, hyperacetylating their rod-specific target genes, accounting for the increased decondensation they observed. But this hyperacetylation comes at a price—the disruption of the normal chromatin architecture of the mature rod nucleus.

The authors suggest that this disruption is the likely explanation for the loss of rod-specific gene transcription. A growing body of research has shown that within the nucleus, the arrangement of chromatin is not random, but is correlated to cell type. Some evidence suggests that highly expressed genes are preferentially located at the nuclear envelope, where they may have better access to the raw materials that sustain rapid transcription. The loss of this structure in the SCA7 mouse retina may prevent the very high levels of transcription of rod-specific genes needed to maintain normal rod function.

This is not likely to be the last word on disease mechanisms in the polyglutamine diseases, a field full of strong and competing hypotheses of pathogenesis. It does, though, highlight the importance of understanding the role of the normal protein, and strengthens the case for altered nuclear architecture as a key event in neurodegeneration.

## 

**Figure:**
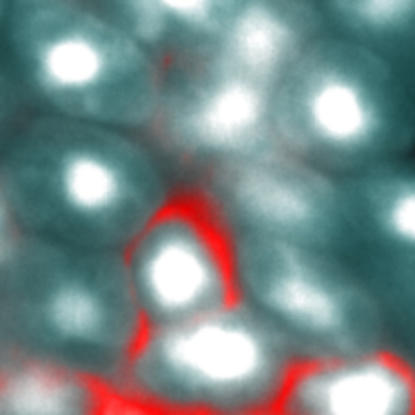
Mutations in ataxin-7 lead to retinal damage by inhibiting the expression of genes involved in chromatin regulation.

